# Genomic recombination between infectious laryngotracheitis vaccine strains occurs under a broad range of infection conditions *in vitro* and *in ovo*

**DOI:** 10.1371/journal.pone.0229082

**Published:** 2020-03-02

**Authors:** Omid Fakhri, Joanne M. Devlin, Glenn F. Browning, Paola K. Vaz, Dulari Thilakarathne, Sang-Won Lee, Carol A. Hartley

**Affiliations:** 1 Asia-Pacific Centre for Animal Health, Faculty of Veterinary and Agricultural Sciences, The University of Melbourne, Parkville, Victoria, Australia; 2 College of Veterinary Medicine, Konkuk University, Seoul, Republic of Korea; Katholieke Universiteit Leuven Rega Institute for Medical Research, BELGIUM

## Abstract

*Gallid alphaherpesvirus 1* causes infectious laryngotracheitis (ILT) in farmed poultry worldwide. Intertypic recombination between vaccine strains of this virus has generated novel and virulent isolates in field conditions. In this study, *in vitro* and *in ovo* systems were co-infected and superinfected under different conditions with two genomically distinct and commonly used ILTV vaccines. The progeny virus populations were examined for the frequency and pattern of recombination events using multi-locus high-resolution melting curve analysis of polymerase chain reaction products. A varied level of recombination (0 to 58.9%) was detected, depending on the infection system (*in ovo* or *in vitro*), viral load, the composition of the inoculum mixture, and the timing and order of infection. Full genome analysis of selected recombinants with different *in vitro* phenotypes identified alterations in coding and non-coding regions. The ability of ILTV vaccines to maintain their capacity to recombine under such varied conditions highlights the significance of recombination in the evolution of this virus and demonstrates the capacity of ILTV vaccines to play a role in the emergence of recombinant viruses.

## Introduction

Herpesviruses are an important cause of disease in a wide range of animal species, including production and companion animals. Infectious laryngotracheitis virus (ILTV; *gallid alphaherpesvirus 1*) is an alphaherpesvirus that causes acute respiratory tract disease in domestic poultry and results in significant economic losses worldwide [1, 2]. The economic losses caused by ILTV are due to the mortalities it causes, as well as its impact on the growth rate of broiler chickens, on egg production in layer farms, and on susceptibility to other respiratory pathogens [1]. Live attenuated vaccines are effective in reducing mortality, clinical signs [2, 3] and controlling the outbreaks of disease [4]. Several recombinant or virally vectored vaccines are also in use or development and include ILTVs with deletions of virulence-related open reading frames (ORFs) [5, 6], and fowl-pox virus (FPV) [7] and turkey herpesvirus (HVT) [8, 9] expressing specific ILTV glycoprotein genes.

Recombination makes a significant contribution to the evolution, diversity and fitness of herpesviruses, particularly in the face of the low rates of point mutation seen in these viruses [10, 11]. Genomic recombination between ILTV strains is common [12–15], and has had a significant impact on currently circulating viruses [15]. *In vitro* and *in vivo* co-infection studies in birds with field strains has revealed recombinational hotspots in the ILTV genome, with specific genomic regions having a higher frequency of recombination events [16]. These studies have shown that recombinant diversity is highest at peak virus replication [16]. Host factors such as vaccination-induced immune responses are also suggested to exert a selective pressure on the emergence of recombinant progeny [17].

The importance of genomic recombination in ILTV was first highlighted with the discovery that two new virulent field strains (class 8 and class 9 ILTV) were recombinants independently derived from distinct attenuated ILTV vaccine strains (Australian origin SA2/A20 and European origin Serva ILTV) [12]. Recombination events between these vaccine strains resulted in viruses of higher fitness that became the predominant field strains in Australia and largely replaced the previously dominant class 2 genotype viruses [15]. Recombination requires a host cell to be simultaneously infected with 2 different virus strains. While simultaneous co-infection of cells or animals can occur under experimental conditions, simultaneous co-infections would not necessarily be expected to occur frequently under field conditions. Instead, some delay between infection with the first and second strain may be more likely. Little is known about the capacity for ILTV strains to allow superinfection, and the role of superinfection in enabling ILTV recombination has not been examined.

The aim of this study was to identify the conditions that lead to recombination between ILTV vaccine strains by performing *in vitro* and *in ovo* co-infection and superinfection experiments under defined conditions. To identify recombinant progeny, we have previously developed a differentiation system based on a panel of high-resolution melting (HRM) curve profiles of the parent isolates [18]. The parental viruses used in this study were the two distinct genetic lineages of ILTV vaccine virus strains, A20 and Serva. In previous studies, these or similar viruses have been found to be the parental viruses from which the virulent recombinant field viruses (class 8 and class 9) have been derived [12].

## Material and method

### Cell and virus culture

Primary chick embryo kidney (CEK) cells were prepared using kidneys harvested from 18-day-old chick embryos as previously described [19]. The use of embryonated eggs was approved by the Animal Ethics Committee of The University of Melbourne (ethics approval number 1814492.1). Monolayers of CEK cells were maintained in Dulbecco’s Modified Eagle Medium (DMEM), 1% v/v foetal bovine serum (FBS), 100 μg ampicillin/mL and 10 μg amphotericin B/mL. All *in vitro* studies were performed with CEK cells, and *in ovo* experiments were performed by inoculation of the chorioallantoic membrane (CAM) of embryonated, specific-pathogen-free (SPF) hybrid white Leghorn-derived chicken eggs (Australian SPF Services, Woodend, Australia).

The vaccine strains NOBILIS^®^ ILT (Serva, GenBank accession number HQ630064) and Poulvac^®^ Laryngo (A20, GenBank accession number JN596963) were plaque purified as described previously [13], propagated on CEK cells and re-typed by PCR-RFLP [20] prior to co-infection experiments. To obtain the high-titred virus stocks required for these studies, each vaccine was passaged twice on the CAM of SPF embryonated eggs [21]. The infected CAMs were extracted and thoroughly homogenised using a mortar and pestle. The homogenates were allowed to sediment for 5 minutes at room temperature to let the larger fragments settle before the supernatants were collected. The CAM supernatants, allantoic fluid (AF) and CEK culture supernatants were aliquoted and stored at -80°C until further use. Viruses were titrated by plaque assay on CEK monolayers [13].

Growth kinetics (multi-step growth curve, entry kinetics and kinetics of plaque development) of Serva and A20 strains of ILTV in CEK cells were measured as described previously [22]. Briefly, for multi-step growth curve, CEK cells in 24-well plates were infected with a MOI of 0.001 and cells and supernatant harvested at the indicated time points. Virus growth was quantified in combined cell lysate and supernatant after DNA extraction (PureLink^®^ Pro 96 viral RNA/DNA purification kit, Invitrogen, USA) using a UL15-specific qPCR, as described by Mahmoudian *et al*. (2011) [23]. The mean and standard deviation of triplicate measurements performed in each of 3 independent experiments were compared by Student’s *t-*test within GraphPad Prism (v. 6.07).

To determine whether Serva and A20 ILTV differ in their entry kinetics, an experiment was performed as described by Mundt *et al*. (2011) [24] with some modifications. Briefly, the CEK monolayers in 6-well plates were chilled to 4°C on ice for 10 minutes, and 500 μL of pre-chilled Serva or A20 inoculum (MOI = 0.0005) were added to each well for adsorption of viral particles on the cell surface. The plates were kept at 37°C (i.e. temperature shift) for 5, 15, 30, 45 and 60 minutes, then the excess inoculum was removed, extracellular virus inactivated with 0.2 M citrate buffer (40 mM citric acid, 10 mM KCl, 135 mM NaCl, pH 3.0) before further washing and the addition of a final 5 mL of 1% w/v methylcellulose in DMEM to each well. The plates were further incubated at 37°C for 72 hours. Viral entry into cells at different time points were calculated by dividing the number of plaques detected at each time point by the number of plaques formed after an incubation period of 60 minutes and reported as percentages. The mean and standard deviation from three independent experiments were then compared using Kruskal-Wallis test followed by Dunn’s multiple comparisons test within GraphPad Prism (v. 6.07).

The capacity of Serva and A20 ILTV to spread from cell to cell was also compared by examining the kinetics of plaque development in CEK cells. The plaque area of 13 to 22 individual plaques photographed at different time points were measured using ImageJ (version 1.50i) [25], and compared using Student’s *t*-test as described previously [22].

Progeny viruses were isolated using ten-fold dilutions of AFs, CAMs, and homogenates of cells and supernatant fluids (SNFs) inoculated onto CEK monolayers in 6-well plates as described previously [18, 22, 26].

### DNA extraction

Nucleic acid extraction was performed using the PureLink^®^ Pro 96 viral RNA/DNA purification kit (Invitrogen, USA) and the Corbett Robotics X-tractor Gene automated vacuum system (Corbett Life Science, Australia). Purified DNA from 200 μL samples was eluted in 200 μL of pyrogen-free water (Milli-Q Integral system, Germany) and stored at -20°C until it was assayed. Pyrogen-free water, rather than the elution buffer supplied with the extraction kit, was used to reduce variation in HRM patterns [27]. For full genome sequencing, viral DNA was extracted from purified nucleocapsids using a High Pure PCR Template Preparation kit (Roche, Germany) according to the manufacturer’s instructions.

### Co-infection and superinfection of CEK monolayers and superinfection of CAMs with a mixture of Serva and A20

For co-infection experiments, CEK monolayers in 6-well tissue culture plates were inoculated with a mixture of the Serva and A20 ILTV strains. Inoculating viruses were mixed prior to infection at the multiplicities of infection (MOI) and ratios shown in Table 1. After 1 hour of incubation at 37°C, the inoculum was removed, and the monolayer was washed three times with 5 mL of PBS, then 3 mL of fresh medium was added. At 48 hours post infection (hpi), extensive cytopathic effect (CPE) was observed, and the cells were scraped into the culture supernatant. Samples collected from these experiments were stored in 1 mL aliquots at -80°C until plaque purification was performed, as described previously [13].

**Table 1:** Summary of inocula used and the samples collected.

Infection model	Total MOI	Serva to A20 ratio	Delay between infections (hours)	First inoculum	Sample collected^a^
CEK cells	10	1:1	0	n/a	Cells & SNF
		1:4	0	n/a	Cells & SNF
		4:1	0	n/a	Cells & SNF
	5	1:1	0	n/a	Cells & SNF
		1:1	2	Serva	Cells & SNF
		1:1	4	Serva	Cells & SNF
		1:1	6	Serva	Cells & SNF
		1:1	8	Serva	Cells & SNF
		1:1	2	A20	Cells & SNF
		1:1	4	A20	Cells & SNF
		1:1	6	A20	Cells & SNF
		1:1	8	A20	Cells & SNF
Embryonated eggs	10^4.5^ pfu/embryo	1:1	0	n/a	CAM
			0	n/a	AF
		1:2	0	n/a	CAM
			0	n/a	AF
		2:1	0	n/a	CAM
			0	n/a	AF
		1:10	0	n/a	CAM
			0	n/a	AF
		10:1	0	n/a	CAM
			0	n/a	AF

^a^ SNF: supernatant fluid, CAM: chorioallantoic membrane, AF: allantoic fluid

In the superinfection study, CEK cells were infected with either the Serva or A20 strain and then, after a range of incubation times, infected with the heterologous virus. After removal of the growth medium from the cells, the first inoculum was added and allowed to adsorb for one hour at 37°C. Prior to removal of the inoculum, extracellular virus was inactivated by washing for one minute in 0.2 M citrate buffer, and then washing several times with growth medium before further incubation of the cells in 3 mL of DMEM until the 2^nd^ inoculation. The 2^nd^ inoculation was performed at 2, 4, 6 or 8 hours after the first (Table 1), using the same procedure as was used for the first inoculation. For the 1:1 simultaneous infection control for this part of the study, the inoculating viruses were premixed prior to inoculation onto the monolayers, and removal of the inoculum and washing with the citrate buffer were performed after one hour as described above. The total MOI used for the superinfection study was 5.

For the *in ovo* co-infection studies, a total of 10^4.5^ plaque forming units (pfu) of the two vaccine strains at varying ratios (Table 1) were inoculated onto each CAM. After inoculation, eggs were incubated at 37°C in a humidified egg incubator (Maro, South Korea) and at 48 hpi the AF and CAM samples were collected separately [28].

### Identifying recombinant progeny viruses using HRM-based multiple-single nucleotide polymorphism (SNP) ILTV genotyping assay and recombination analysis

Any detection of recombinant progeny was used as a readout of co-infection or superinfection of cells. In order to detect recombinant viruses, individual viruses were cultured and plaque purified. In all experiments, progeny viruses were plaque purified on CEK monolayers in 6-well plates as previously described [13]. At least 20 progeny viruses were randomly picked per infection condition and plaque purified three times prior to recombination analysis using the HRM panel.

The analysis of isolates was performed using a technique we have described previously [18]. Briefly, the DNA extracted from plaque purified isolates was tested using a panel of 6 PCR-HRM assays targeting 6 sites (UL52, UL27, UL36, UL8, IR and US4) that differ between the A20 and Serva genomes. To confidently assign genotype to the progeny viruses, this assay is used to examine only plaque purified populations of virus. Therefore, any that was not purified as expected and showed a mixed SNP/HRM profile in any of the 6 sites were considered as a mixed population and were excluded from further analyses. Following the PCRs, a 6-character genotype pattern code was applied to each isolate as an identifier. When the HRM curve of an isolate matched Serva in a region, the letter A was used as the identifier, therefore a pure Serva parent genotype pattern code is AAAAAA. If the HRM curve matched A20 the letter B was incorporated into the genotype pattern code, therefore a pure A20 parent genotype pattern code is BBBBBB. Recombinant viruses were those identified as having a mixture of Serva and A20 genomic regions, with a mixture of A and B regions in their 6-character genotype pattern code (e.g. ABBAAB). All possible genotype pattern codes (64 codes) were also given a number (genotype code 0 to 63) for ease of referencing [18].

### Full genome sequencing

One representative isolate from each genotype pattern code (33 patterns isolated in total) was screened to look for possible variations in their plaque sizes on CEK cells. Plaque areas were measured using ImageJ (version 1.50i) [25] calibrated using a stage micrometre [22]. Eight isolates were selected for full genome analysis based on their HRM genotype and their plaque size. These isolates were further cultured by passage on CEK cells in 175 cm^2^ tissue culture flasks with 50 mL of growth medium. The viruses were harvested 72 hours after infection, when extensive CPE was observed. The cells were scraped into the growth medium, homogenized by pipetting and stored in 50 mL aliquots at -80°C. A freeze-and-thaw step was included in order to maximise the release of viruses from cell debris. Viral nucleocapsids were purified from the lysate as described by Atherton *et al*. [29].

#### Library preparation and sequencing

Extracted DNA samples were sent to the Australian Genome Research Facility (AGRF) for library preparation and whole-genome sequencing. The libraries were prepared using Nextera XT Index kits with no insert size selection. The paired-end sequences were determined using an Illumina MiSeq sequencing machine with a Nano flow cell and 300 cycles. The total number of reads and the read lengths are shown in Table 2.

**Table 2:** Full genome reads and assembly metrics for the 8 isolates selected for full genome sequenced in this study.

Isolate number	Genome size (bp)	Total reads	Mapped reads (after trimming)	Breadth of coverage of reference* (%)	Mean depth of coverage (number of reads)
29	152,643	327038	23,405	99.90	21.0
109	152,627	309310	27,202	99.90	24.5
138	152,660	338586	40,504	99.90	36.6
157	152,663	329770	70,534	99.98	63.6
237	152,713	273274	27,759	99.90	24.7
238	152,717	317580	33,116	99.99	29.8
319	152,686	301232	38,651	99.90	34.0
358	152,600	314154	88,048	100.00	79.3

* Serva ILTV strain, GenBank accession number HQ630064, was used as the reference

#### Genome assembly of progeny viruses

Viral genome assembly using the Illumina read data was performed using the Geneious 11.1.4 sequence analysis package [30]. The paired-end short reads generated for each isolate were trimmed with a quality threshold Phred score of 30 and the Nextera adapters were removed using the BBDuk 37.64 tool (Brian Bushnell within Geneious 11.1.4). The trimmed reads were then assembled by mapping the raw reads to the published Serva ILTV genome (Genbank accession HQ630064) as a guide using Geneious Mapper in the “Map to Reference” tool with “Medium Sensitivity” and up to 5 reiterations for fine tuning the assembly. Following the assembly, consensus sequences were generated using read quality as threshold. The total number of reads, mapped reads, breadth of coverage, average read length, and mean depth of coverage for each isolate are shown in Table 2. The full genome sequences of these isolates are available in the GenBank database under the accession numbers (Table 3).

**Table 3:** Comparison of plaque areas in CEK cells of 8 recombinant viruses at 48 hpi with those of Serva and A20 parental strains.

Strain or isolate	Genotype code	Genotype pattern code	Mean plaque area ± SD (mm^2^) ^a^	GenBank accession number
A20	0	BBBBBB	0.72 ± 0.21	JN596963
Serva	63	AAAAAA	0.58 ± 0.25	HQ630064
29	57	AAABBA	0.10 ± 0.03 ^#^	MK894996
109	57	AAABBA	1.06 ± 0.34 ^#^^,^ ^†^	MK894997
138	57	AAABBA	1.17 ± 0.46 ^#^^,^ ^†^	MK894998
157	39	ABBAAA	1.34 ± 0.44 ^#^	MK894999
237	17	BABBBA	0.88 ± 0.35 ^‡^	MK895000
238	24	BAABBB	0.17 ± 0.07 ^#^	MK895001
319	0	BBBBBB	0.36 ± 0.22 ^¥^	MK895002
358	63	AAAAAA	0.56 ± 0.19	MK895003

^a^ Comparisons were performed using a one-way ANOVA followed by Tukey’s test for pairwise comparisons within GraphPad Prism (v. 6.07); We considered *p* values of <0.05 to be statistically significant

^#^ Significant difference with Serva and A20

^†^ Significant difference with #29

^‡^ Significant difference with Serva

^¥^ Significant difference with A20

#### Identification of recombination events

After aligning the recombinant whole genome sequences to the parental sequences, recombination events were initially detected by visually/manually reviewing the sequences for known SNPs within the Geneious viewer tool, and the exchanged regions of the genome were marked as Serva-like or A20-like. In addition, the 8 viruses sequenced in this study were individually aligned to the Serva and A20 reference genomes using the MAFFT aligner [31] within Geneious and sequence similarity plots of these sequence trios were generated using SimPlot version 3.5.1 [32]. The recombination events detected in these sequence trios were then verified using the Recombination Detection Program version 4 (RDP4, version beta 4.95) [33] (S1 Table). In SimPlot, the sliding window size was set to 6000 bp to scan the genome in 200 bp steps and F84 (maximum likelihood) was used as the distance model. A manual reviewing step was included to ensure that the recombination detection algorithms had not overlooked small fragment exchanges due to the high sequence identity (99.2%) between the parental strains. For the visual/manual detection of recombination in the aligned sequences, the full genome sequence of each recombinant was aligned to those of Serva and A20, and the disagreements were highlighted after setting the Serva genome as the reference sequence. Following this, the regions in recombinants that contained Serva-like SNPs were annotated as Serva-like, and those that contained A20-like SNPs were annotated as A20-like.

## Results

### Growth kinetics of the Serva and A20 ILTV strains

**Fig 1:**
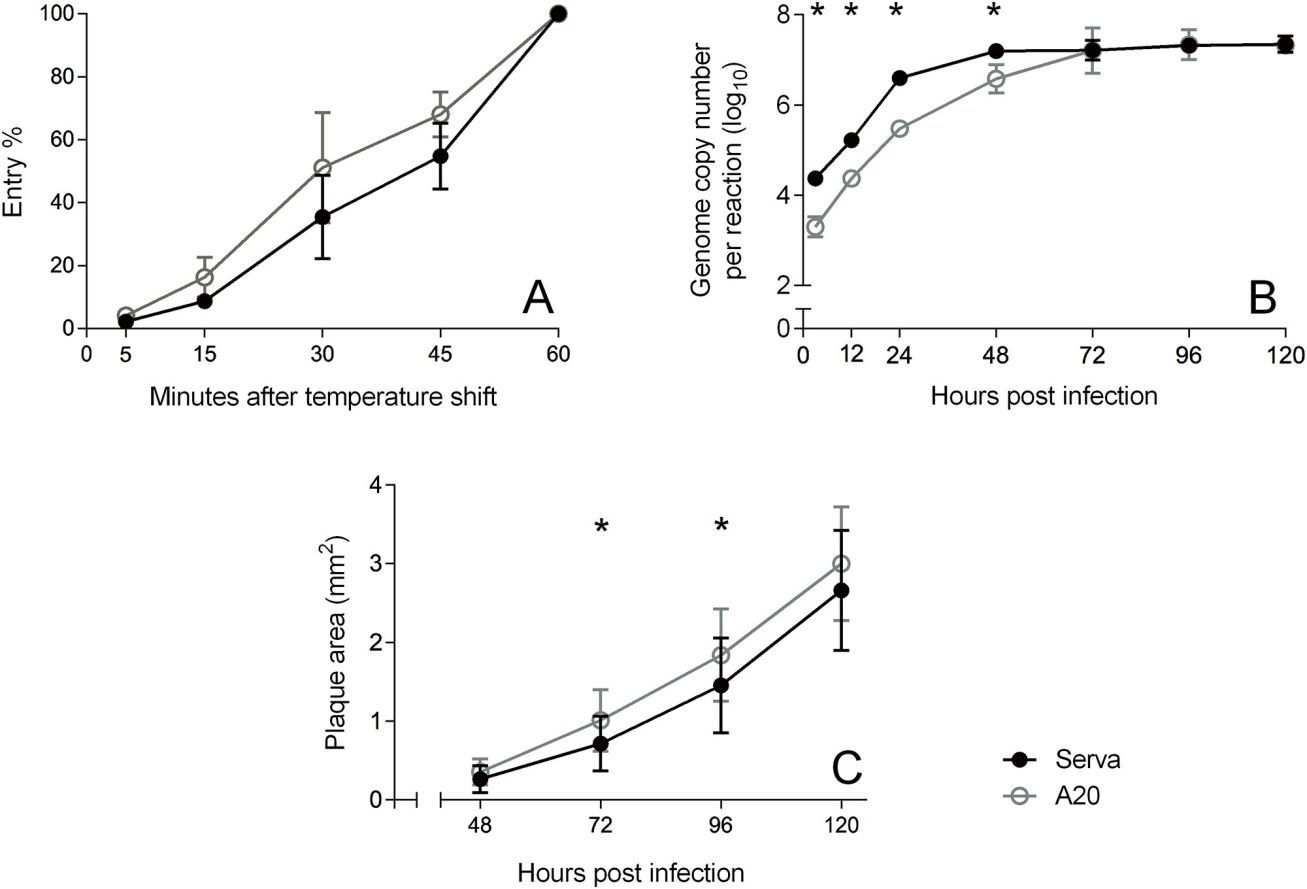
Growth kinetics of Serva (closed circles) and A20 (open circles) strains of ILTV in CEK cells. (a) Entry kinetics of Serva and A20 in CEK cells. The mean and standard deviation from 3 independent experiments are shown. No significant difference between the two different viruses was detected at any time point (b) Multi-step growth curve of Serva and A20 in CEK cells infected with a MOI of 0.001. The mean and standard deviation of triplicate measurements performed in each of 3 independent experiments are shown; * indicates *p* < 0.05 (Student’s *t*-test). (c) Kinetics of plaque development by the Serva and A20 strains of ILTV in CEK cells. The mean and standard deviation of the plaque area (mm^2^) of 13 to 22 individual plaques at each time point are shown; * indicates *p* < 0.05 (Student’s *t*-test).

Given the close association between replication and recombination [16], the *in vitro* growth characteristics of both parental viruses used in this study were compared. Both the Serva and A20 strains had similar entry kinetics into CEK cells, with no significant difference between them at any time point (Fig 1A).

In the multistep growth curve comparison, both A20 and Serva plateaued to similar maximal titres at 48 to 72 hpi, although cultures of Serva had significantly higher genome concentrations (*p* < 0.05, Student’s *t*-test) than the A20 strain in the exponential growth phase (Fig 1B). A20 produced significantly larger plaques (*p* < 0.05, Student’s *t*-test) than Serva at 72 and 96 hpi, although the plaque sizes did not differ significantly at 48 hpi and 120 hpi (Fig 1C).

### Recombinant progeny are produced under a broad range of infection conditions

**Fig 2:**
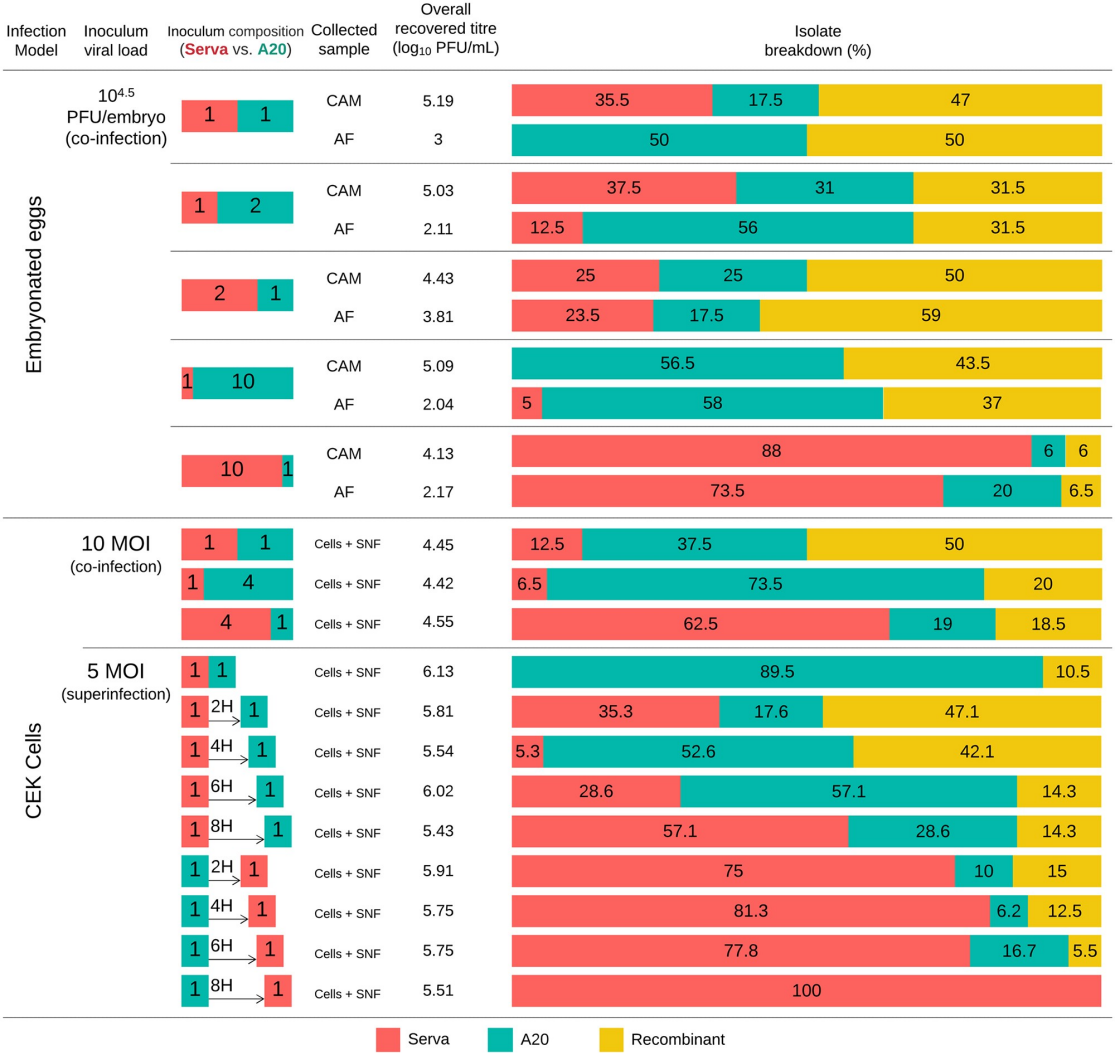
Summary of results from co-infection and superinfections *in ovo* and in CEK cultures. Proportions of parental strains or recombinants in both the inoculum and progeny viruses are shown as red (Serva), green (A20), or yellow (recombinant) blocks. The isolates from co-infection/superinfection experiments on CEK cells were obtained from a combination of the cell lysate and the supernatant fluid (Cell + SNF) from the infected CEK monolayers. The isolates from the co-infection experiment in embryonated eggs were obtained from CAM homogenates or AF, which were collected separately. The overall titre of virus recovered from each infection condition is also shown.

The titres of ILTV recovered from co-infected CEK cells, superinfected CEK cells, CAMs and AFs are shown in Fig 2. A summary of these results for co-infection/superinfection of CEK monolayers and eggs is shown in Fig 2, with the proportion of recombinants calculated by considering any progeny that did not have a 6-character genotype pattern code matching the parental strains (AAAAAA or BBBBBB) as recombinant.

### *In ovo* co-infections

**Fig 3:**
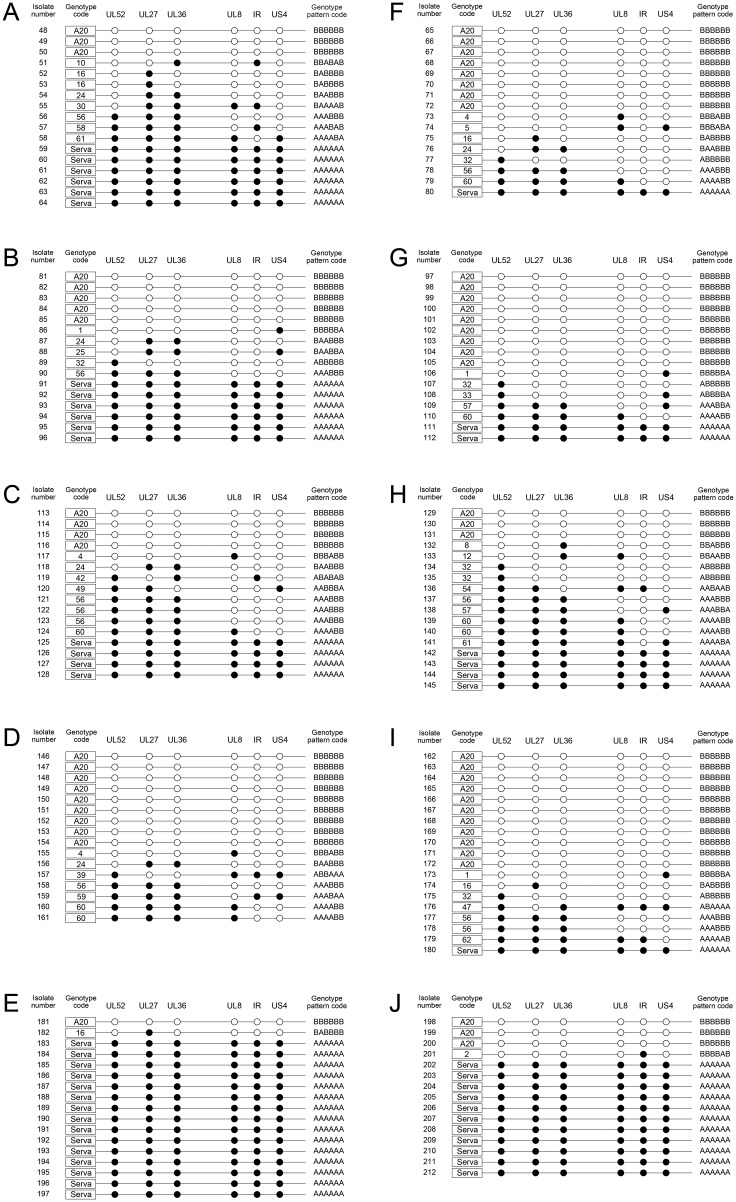
Schematic view of SNP distribution, showing the composition of the progeny population collected from the CAM (left column = A, B, C, D and E) and AF (right column = F, G, H, I and J) of co-infected chicken embryos 72 hours after inoculation. Five eggs were inoculated per group and the viruses were isolated from 3 or 4 eggs (pooled samples) that did not show any signs of embryonic death. The panels represent each ratio of infecting parent viruses (Serva:A20) as 1:1 (A, F), 1:2 (B, G), 2:1 (C, H), 1:10 (D, I) or 10:1 (E, J). The closed circles indicate Serva-like SNPs and open circles indicate A20-like SNPs. Each row represents a virus that was genotyped as A20, Serva, or a recombinant with a unique genotype code (1 to 62) for each genotype pattern code.

In co-infected eggs, recombinant progeny were detected under all conditions tested. In these experiments, the ratio of parental viruses in the inoculum varied from equal proportions to a 10:1 excess of one of the parental strains. Between 47 and 50% of the progeny of the 1:1 co-infections were recombinants, and a 2:1 ratio of the parental viruses still resulted in a high proportion of recombinant progeny (31.5–59%). However, when a 10:1 ratio of the parental viruses was inoculated, differences in the proportion of recombinant progeny were seen, depending on which parental virus was included at a higher concentration. In eggs that were inoculated with 10 times more Serva than A20, a considerable reduction in the proportion of recombinant progeny was seen (≤ 6.5% recombinants), while when 10 times more A20 was inoculated, a reduction in the proportion of recombinants was not detected (37–43.5% recombinants). The ratio of recombinants to parental viruses (Serva and A20 combined) was similar regardless of the site of recovery (CAM or AF) (Fig 2). The genotype pattern codes of the recombinant progeny detected in these co-infected eggs are shown in Fig 3.

### Co-infection of CEK cells

**Fig 4:**
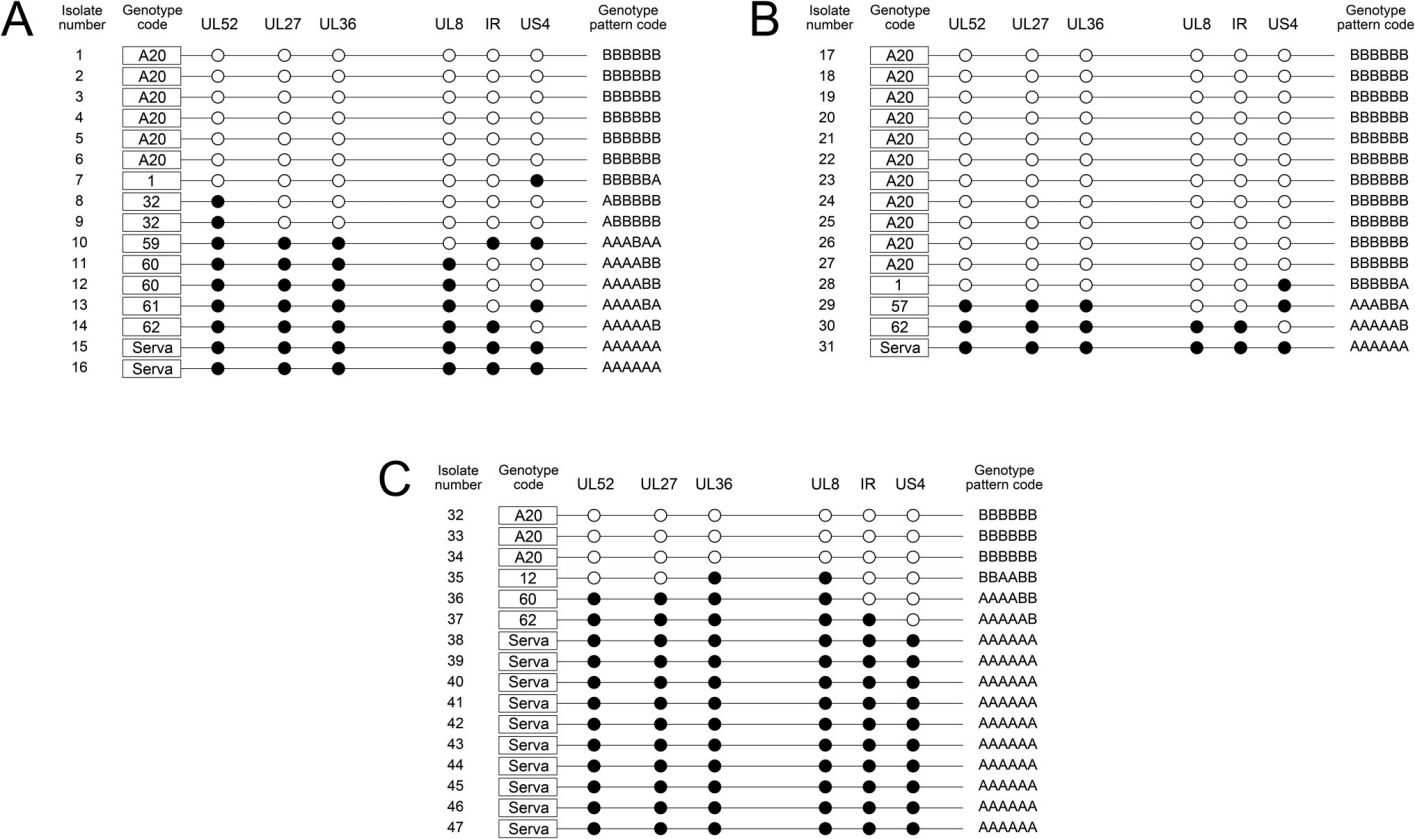
The composition of the progeny population collected from CEK cells co-infected with A20 and Serva ILTV at a total MOI of 10. The distribution of Serva-like (closed circles) and A20-like (open circles) SNPs in each isolate is shown. The isolates were collected from (A) 1:1 co-infection, (B) 1:4 (1 part Serva, 4 parts A20) co-infection, and (C) 4:1 (4 parts Serva, 1 part A20) co-infection.

The influence of the ratio of the parental viruses on the proportion of parent and recombinant viruses after simultaneous co-infection was also examined in CEK cells. Recombinant progeny were detected in all experiments, regardless of the ratio of each infecting parental strain. In these experiments, the highest proportion of recombinant progeny (50%) was detected after co-infection with a 1:1 ratio of the two viruses, and the proportion decreased when the ratio of parental viruses was less balanced (4:1 or 1:4). Regardless of which parental virus was in excess, the proportion of recombinant progeny was similar at 18.5–20% (Fig 2). The genomic patterns of the recombinant progeny detected after CEK co-infection are shown in Fig 4.

### Superinfection of CEK cells

A co-infection control was included in this experiment to enable a direct comparison (Fig 2). The profile of the progeny viruses from the co-infection control was notable for the absence of any detectable Serva strain among the progeny, with 89.5% of the progeny the A20 parent and 10.5% recombinants.

**Fig 5:**
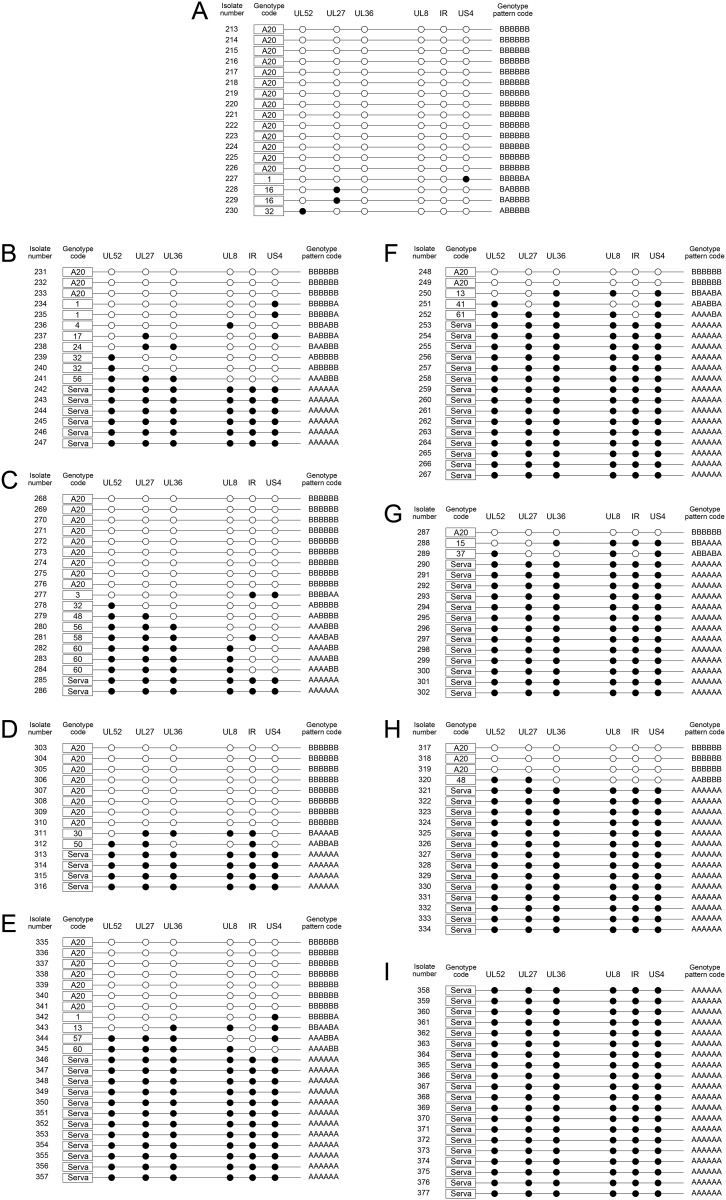
Schematic view of SNP distribution showing the composition of the progeny population collected from CEK cells (A) co-infected at a MOI of 5 with Serva and A20, or (B-I) superinfected with either Serva or A20. The left panels (B, C, D, E) show primary infection with Serva, while the right panels (F, G, H, I) show primary infection with A20. The incubation period between primary and secondary infection was 2 (B, F), 4 (C, G), 6 (D, H) or 8 (E, I) hours. The samples were collected at 72 hpi. The closed circles indicate Serva-like SNPs and open circles indicate A20-like SNPs. Each row represents a virus that was genotyped as A20, Serva, or a recombinant, with a unique genotype code number (1 to 62) for each genotype pattern code.

The MOI of 5 used in these experiments should have been sufficient to infect >95% of the cells according to the predictions based on the Poisson distribution [34], so the presence of any genomic region derived from the second virus was considered an indication that superinfection had occurred. The results of the superinfection experiments showed that ILTV-infected cells can be superinfected with a second ILTV strain. The second infecting strain was detected among the progeny virions in all cases, even when the second inoculation occurred 8 hours after the first (Fig 2). Recombinant progeny were detected in all superinfections, except when cells were superinfected with the Serva strain eight hours after primary inoculation with A20. However, as all the progeny virions were the second infecting strain (Serva), the observations were still consistent with successful superinfection. The highest proportion of recombinant progeny was detected when there was a 2- to 4-hour gap between infections, with Serva as the initial infecting virus (42.1 to 47.1%). The genomic patterns of the recombinant progeny detected after CEK co-infection are shown in Fig 5.

### Genotype pattern of the recombinant progeny

**Fig 6:**
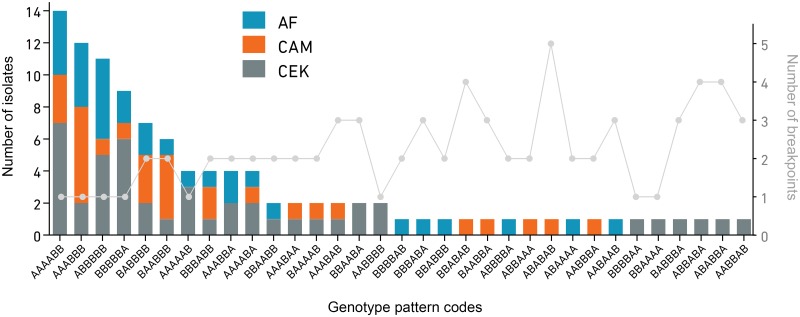
Number of isolates with each genotype pattern code (left y axis) and the origin of each isolate, together with the number of recombination breakpoints on the right y axis. Isolates resulting from multiple recombination events were obtained less frequently. The negative correlation between the number of recombination events in a recombinant virus and the abundance of those recombinants in samples was statistically significant (Spearman’s correlation, *r*_*s*_ = -0.55, 95% confidence interval = -0.7579 to -0.2490, two-tailed *p* value = 0.0008).

With few exceptions (Figs 3E, 3J and 5H), different infection conditions resulted in isolation of recombinant viruses representing more than one genotype. The recombinants detected in these studies had 33 unique genotypes based on the HRM analyses (Fig 6). Of these 33 genotypes, 17 were detected only once, while 16 were isolated on more than one occasion, either within the same group or from different co-infection conditions. In CEK cells, the most abundant recombinant genotype pattern among the progeny was genotype code 60 (AAAABB), while in eggs genotype code 56 (AAABBB) was the most abundant. In general, isolates derived from multiple recombination events were recovered less frequently than those that were consistent with a single recombination event.

### Full genome sequencing of progeny isolates

Eight progeny viruses were selected for full genome sequence analysis to more finely map the recombination events. Viruses were chosen for sequencing based on their genotype pattern code and differences in plaque sizes in CEK cells (Table 3). These included 3 isolates with the genotype pattern code AAABBA (isolates 29, 109 and 138) that had differing plaque sizes and 4 isolates with different genotype pattern codes and a range of plaques sizes derived from the superinfection experiments (isolates 237, 238, 319 and 358). Two isolates were also selected that had genotype pattern codes identical to the parental strains (isolate 319 was A20-like and isolate 358 was Serva-like by HRM). Isolate 319 formed significantly smaller plaques than the A20 parent, but the Serva-like candidate 358 formed plaques that were similar in size to those formed by the Serva strain (Table 3).

The full genome sequences of the 8 isolates were mostly consistent with results from the HRM genotyping assay, with each of the 8 viruses having the same SNPs that were identified by HRM, with one exception.

#### Isolates 29, 109 and 138 (genotype pattern code AAABBA)

**Fig 7:**
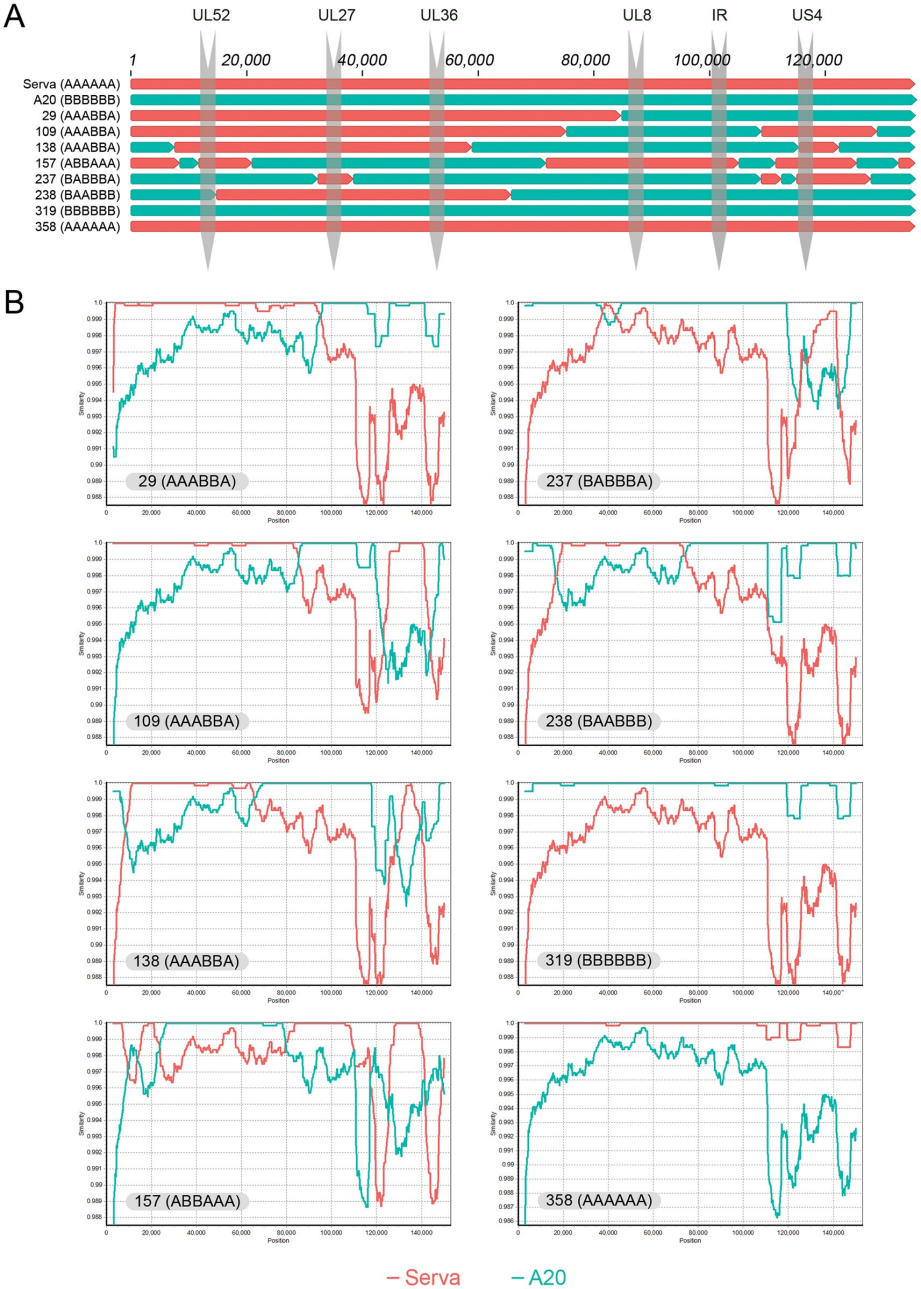
Schematic view of the genomic composition of the isolates sequenced in this study (A) and sequence similarity plots (B) of isolate #29, #109, #138, #157, #237, #238, #319 and #358 with those of Serva and A20. The plots were generated using SimPlot v3.5.1 set to scan the sequences with 6000 bp sliding windows, which were moved across the alignments in 200 bp steps.

The US4 region of isolate 29 was Serva-like in the full genome sequence analysis, not A20-like, as detected by the HRM analysis. This event was miscategorised because of a different change in the amplicon that resulted in a change in the *T*_m_ of the amplicon to one similar to that seen with amplicons from A20 (Table 4, Isolate 29, position 131365). Therefore, while isolate 29 was classified as AAABBA by HRM analysis, the correct genotype code for this isolate based on the full genome sequence would be AAABBB (see top panel, Fig 7). Similarity plot analyses of the full genome sequences of these isolates detected one further recombination event in isolates 29 and 138, but not in isolate 109 (Fig 7). Isolate 29 has a significantly smaller plaque size than the 2 other isolates (109, 138) with the AAABBA genotype pattern code (Table 3) and compared to the parental sequences, each of these isolates had a range of SNPs in the core genes that resulted in disruptive frame shifts and amino acid changes (Table 4).

**Table 4:** Summary of polymorphisms in ILTV isolates sequenced in this study.

Isolate number (genotype pattern code)	Origin of isolate	Original position in sequenced isolate	Gene involved	Polymorphism type	Nucleotide/codon change	Amino acid change
29 (AAABBA)	CEK cells co-infected with 10 MOI (1:4)	10809	UL54	SNP (transition)	ATA–ATG	I–M
		17348	UL51	SNP (transition)	CAC–CAT	None
		55545	UL36	SNP (transition)	CGC–CAC	R–H
		69827	UL39	SNP (transversion)	AAA–AAC	K–N
		75368	UL43	SNP (transition)	ACA–ATA	T–I
		79996	UL20	SNP (transition)	GAT–GAC	None
		131365	US4	SNP (transition)	TCT–CCT	S–P
109 (AAABBA)	AF of 1:2 co-infection	58644	UL36	SNP (transition)	AAG–AAA	None
		109522	UL0	Insertion (tandem repeat)	(C)6–(C)7	Frame Shift
138 (AAABBA)	AF of 2:1 co-infection	58705	UL36	SNP (transition)	AAG–AAA	None
		59226	UL36	SNP (transition)	GTG–ATG	V–M
		120488	Intergenic	SNP (transversion)	A–C	n/a
		131933	US4	SNP (transition)	ACG–GCG	T–A
157 (ABBAAA)	CAM of 1:10 co-infection	15128	UL52	SNP (transition)	TGC–TGT	None
		72497	UL41	SNP (transversion)	GCA–GAA	A–E
237 (BABBBA)	CEK cells re-inoculated w/ A20 2 hours after Serva inoculation	109582	UL0	Insertion (tandem repeat)	(C)6–(C)7	Frame Shift
238 (BAABBB)	CEK cells re-inoculated w/ A20 2 hours after Serva inoculation	26366	ORFB CDS	SNP (transversion)	CAA–CAT	Q–H
319 (BBBBBB)	CEK cells re-inoculated w/ Serva 6 hours after A20 inoculation	21656	UL46	SNP (transition)	GCC–GCT	None
		90066	UL15	SNP (transition)	ACA–GCA	T–A

Each recombinant isolate was aligned with the Serva and A20 genomes and variations* unique to the isolate, and not present in either parent, are shown.

* Variations from A20 and Serva genomes were called using “Find variations/SNPs” tool within Geneious (version 11.1.4).

#### Isolates 157, 237, 238

Full genome sequencing was consistent with the HRM recombinant genotyping of these viruses and no further recombination events were detected by Simplot analysis of these recombinant viruses. A small number of changes were detected in these isolates compared to the parental sequences (Table 4 and Fig 7).

#### Isolate 319

This isolate was sequenced because it had the same HRM genotype pattern code as the A20 parent but formed significantly smaller plaques in CEK cells. The full genome sequence was consistent with the HRM genotyping, and the genome sequence of isolate 319 was identical to that of A20, except for 2 SNPs. Only one of these SNPs resulted in a nonsynonymous mutation, from T_195_ to A_195_ in the UL15 gene product. This encodes the DNA packaging terminase subunit 1, which is the ATPase domain of the large terminase subunit [32]. While T_195_ is not a conserved amino acid among all herpesvirus UL15 homologues, it is conserved among the UL15 homologues in each of the 40 full-genome sequences of ILTV currently available in public repositories.

## Discussion

Recombination is a common event in ILTV infections [12, 14, 16, 17] and the results of this study support and extend previous studies by demonstrating the resilience of ILTV recombination under a broad range of infection conditions. Here we have shown that the A20 and Serva vaccine strains can recombine in both CEK cells and chicken embryos, generating intertypic recombinants under a wide range of conditions. Recombination was detected whether or not there was an imbalance in the ratio of parental viruses in both *in vitro* and *in ovo* co-infection models and could also be detected when there was a significant (up to 8 hour) delay between the primary and secondary infections. These findings are consistent with prior studies showing the production of a high proportion of recombinant progeny under both laboratory and field conditions by a range of different ILTV strains [12, 16].

In most cases, optimal conditions for recombination appeared to occur when equal amounts of both parental viruses were inoculated, with a delay after inoculation of the A20 strain of 4 hours or less. Under these conditions, 42 to 50% of the progeny viruses were genotyped as recombinants. The ratio of viruses that were typed as parental strains in the progeny virus population rarely reflected the composition of inoculum. For instance, no Serva-like virus was isolated from the samples that were collected from the AF of eggs inoculated with equal amounts of the Serva and A20 strains. Analysis of a much larger number of progeny viruses would be required to determine if any Serva parent could be detected among this population, however the detection of recombinants shows that while infection by Serva has occurred, this appears to have been outcompeted by A20 and the recombinant viruses.

The presence of any recombinant viruses is indicative of co-infection or superinfection by the second strain. In this study, both parental strains clearly had considerable capacity to superinfect CEK cells and superinfection occur up to 8 hours after the first virus had been inoculated, for either of the viruses. When there was an 8-hour delay and the Serva strain was the superinfecting virus, no recombinant viruses were detected and Serva was the only type detected in the progeny. Alphaherpesviruses have been shown to be able to prevent a cell being infected by a second virus (superinfection exclusion) [35], but this has not been studied previously for ILTV. Superinfection exclusion is considered to occur mainly through a receptor interference mechanism involving the expression of glycoprotein D (gD) [36–38], but has also been shown to occur through gD-independent mechanisms [39]. In herpes simplex virus 1 and pseudorabies virus (PRV), superinfection exclusion occurs by 2 hours after infection and does not coincide with the expression of gD in infected cells. The timing of gD translation in ILTV has not been studied, but transcripts are detected as early as 1 hpi and it has been classified as an early/late gene [40]. The coding sequence of gD in Serva differs from that of A20 by seven SNPs. Six of these 7 SNPs are non-synonymous. The high number of non-synonymous mutations in this gene, despite the similarities in superinfection exclusion by these two strains seen in the study described here, might indicate that this gene does not have a crucial role in controlling superinfection. In this study, superinfection occurred up to 8 hours after primary infection, so it appears that superinfection exclusion is unlikely be a significant factor in ILTV infection. Other alphaherpesviruses that are controlled using live attenuated vaccines (e.g. PRV and bovine herpesvirus-1) also have the capacity to establish superinfection *in vitro* or *in vivo*, and to generate recombinant viruses that cannot be effectively controlled using the conventionally used attenuated vaccines [41–44].

The generation of recombinant ILTV viruses in the field has resulted in the spread of viruses with increased fitness [12, 14, 15]. The range of infection conditions in this study generated recombinant viruses with 33 unique genotype pattern codes. The analysis of the genotype pattern codes highlighted the strong negative correlation between the number of recombination events in an isolate and the abundance of those isolates. As shown in Fig 6, the most abundant genotype pattern codes were those that resulted from a single recombination event (single recombinants). Isolates with genotype codes indicating multiple recombination events were rarely isolated more than once, consistent with results from prior recombination studies on other ILTV strains [16], and on bovine herpesviruses [45].

Recombination could help to maintain relatively high diversity among populations of ILTV in different host environments. Full-genome sequence analysis was used to analyse recombination events in finer detail. These analyses supported the results of the HRM assay and identified additional variations that were not detected in the HRM assay (Table 4). These variations included SNPs, insertions/deletions and substitutions. Some of these led to protein sequence changes (Table 4), which could potentially affect the growth of the virus at most stages of viral replication cycle. The isolates with an identical HRM assay type (genotype code 57) had significantly different plaque sizes (Table 3), which may be a result of multiple point mutation differences between these isolates, as listed in Table 4. In addition, one isolate that was detected as a parent-like (A20-like) virus (isolate 319) had a significantly different plaque size compared to the reference A20 strain. This isolate had only one non-synonymous change, in a region that codes for a protein involved in packaging of DNA (UL15).

The existence of field viruses that are recombinants between similar vaccine strains [12] is consistent with the ability of ILTV to establish co-infection and superinfection *in vitro* and *in ovo*, although this has yet to be tested experimentally in chickens. This resilience further establishes the importance of strict biosecurity and the application of good vaccination practice on poultry farms, in particular the importance of use of only one type of vaccine per flock. The finding that a relatively high proportion of commercial layer birds are shedding ILTV vaccine at any given time [46] is a strong indication that ILTV vaccine strains will be important in the evolution of ILTV in the field. Recombination could occur after infection of vaccinated chickens with wild-type strains of ILTV following a biosecurity breach; or the infection of vaccinated chickens with reactivated latent ILTV following the stress of re-vaccination. The use of multiple vaccines could further enhance the genomic diversification of ILTV in these scenarios. Future investigations are required to help determine the behaviour of ILTV when infecting the natural host.

## Supporting information

S1 Table Recombination breakpoint analysis of ILTV isolates sequenced in this study, generated by RDP4 software. (DOCX)
