# Supplementary material for: Genomic recombination between infectious laryngotracheitis vaccine strains occurs under a broad range of infection conditions in vitro and in ovo
Source: PLoS One. 2020 Mar 2;15(3):e0229082. doi: 10.1371/journal.pone.0229082 (PMC7051062; doi:10.1371/journal.pone.0229082)
Supplement: S1 Table — (DOCX) [file pone.0229082.s001.docx]

**S1 Table.** **Recombination breakpoint analysis of ILTV isolates sequenced in this study, generated by RDP4 software**

| **Recombination event number** | **Isolate number** | **Exchanged segment position** | | ***p* value of methods by which breakpoint was detected in RDP4** | | | | | | | | |
| --- | --- | --- | --- | --- | --- | --- | --- | --- | --- | --- | --- | --- |
|  |  | **In alignment** | **Relative to Serva** |  |  |  |  |  |  |  |  |  |
|  |  |  |  | **RDP** | **GENECONV** | **Bootscan** | **MaxChi** | **Chimaera** | **SiSscan** | **PhylPro** | **LARD** | **3Seq** |
| 1 | 29 | 928–94394 | 909–94124 | 8.21E-98 | 4.18E-97 | 1.63E-34 | 1.73E-42 | 5.42E-43 | NS | NS | NS | 1.11E-16 |
| 2 | 109 | 84978–122578 | 84709–122265 | 2.53E-62 | 1.05E-63 | 4.70E-55 | 3.94E-34 | 8.45E-34 | NS | NS | NS | 1.11E-16 |
| 3 | 109 | 144702–152025 | 144332–151616 | 5.61E-37 | 1.52E-49 | 8.66E-46 | 6.48E-17 | 3.12E-16 | NS | NS | NS | 1.11E-16 |
| 4 | 138 | 8766–66662 | 8500–66393 | 1.81E-67 | 1.15E-66 | 1.69E-62 | 1.04E-30 | 1.76E-30 | NS | NS | NS | 1.11E-16 |
| 5 | 138 | 130265–138154 | 129939–137798 | 1.45E-45 | 1.32E-45 | 1.48E-45 | 1.33E-13 | 1.71E-13 | NS | NS | NS | 1.11E-16 |
| 6 | 157 | 23846–81159 | 23571–80881 | 4.79E-44 | 1.68E-39 | 4.76E-44 | 6.48E-23 | 4.49E-23 | NS | NS | NS | 1.11E-16 |
| 7 | 157 | 142414–149663 | 142040–149266 | 8.01E-22 | 7.60E-17 | 3.88E-18 | 1.03E-18 | 8.38E-18 | NS | NS | NS | 1.11E-16 |
| 8 | 157 | 117468–125491 | 117184–125166 | 4.88E-30 | 7.45E-22 | 2.29E-16 | 1.20E-19 | 8.08E-18 | NS | NS | NS | 1.11E-16 |
| 9 | 157 | 9787–13504 | 9512–13229 | 2.90E-23 | 6.49E-23 | 2.91E-23 | 1.26E-08 | 1.24E-08 | NS | NS | NS | 8.77E-15 |
| 10 | 237 | 129824–144268 | 129497–143901 | 5.21E-51 | 1.20E-48 | 1.64E-18 | 6.70E-20 | 5.15E-20 | NS | NS | NS | 2.22E-16 |
| 11 | 237 | 122974–126790 | 122660–126466 | 8.54E-25 | 4.42E-24 | 1.02E-21 | 6.18E-08 | 6.69E-07 | NS | NS | NS | 3.52E-14 |
| 12 | 237 | 36752–43529 | 36483–43260 | NS | 6.12E-08 | 6.45E-09 | 4.69E-02 | 4.51E-02 | NS | NS | NS | 3.81E-04 |
| 13 | 238 | 16966–74234 | 16700–73965 | 4.80E-75 | 4.43E-76 | 1.14E-16 | 2.56E-27 | 3.41E-27 | NS | NS | NS | 2.22E-16 |
| 14 | 238 | 113903–114416 | 113631–114144 | 6.11E-32 | 1.10E-30 | 6.11E-32 | 2.11E-10 | 2.03E-10 | NS | NS | NS | 2.22E-16 |

***NS*** No significant *p* value was recorded for this recombination event using this method. The highest acceptable *p* value was set to 0.05 and the sizes of the scanning windows were set to 30-200 bases depending on the method used.
